# The burden of iatrogenic obstetric fistulas in Sub-Saharan Africa: Systematic review and meta-analysis protocol

**DOI:** 10.1371/journal.pone.0302529

**Published:** 2024-08-26

**Authors:** Mercy M. Imakando, Ernest Maya, David Owiredu, Mercy W. Monde, Choolwe Jacobs, Isaac Fwemba, Kwadwo Owusu Akuffo, Anthony Danso-Appiah

**Affiliations:** 1 Department of Population, Family and Reproductive Health, School of Public Health, University of Ghana, Legon, Accra, Ghana; 2 Department of Obstetrics and Gynaecology, Women and Newborn Hospital, University Teaching Hospitals, Lusaka, Zambia; 3 Centre for Evidence Synthesis and Policy, University of Ghana, Accra, Ghana; 4 Medical Library, University of Zambia, Lusaka, Zambia; 5 Department of Epidemiology and Biostatistics, School of Public Health, University of Zambia, Lusaka, Zambia; 6 Department of Optometry and Visual Science, College of Science, Kwame Nkrumah University of Science and Technology, Kumasi, Ghana; 7 Department of Epidemiology and Disease Control, School of Public Health, University of Ghana, Legon, Accra, Ghana; National Academy of Medical Sciences, NEPAL

## Abstract

**Background:**

Obstetric fistulas are abnormal open connection(s) between the vagina and the urinary tract or the rectum resulting from tragic injuries sustained by mothers during childbirth that lead to urine and/or faecal incontinence. Due to the rapidly growing middle class in sub-Saharan Africa (SSA) and the corresponding quest for hospital delivery and caesarean section, surgery-related (iatrogenic) obstetric fistulas are on the rise. Worryingly, there is scanty data on surgery-related fistulas. This review aims to collate empirical evidence on the magnitude of iatrogenic obstetric fistulas in SSA, generate country-specific data and explore factors that influence obstetric surgery-related fistulas.

**Methods:**

All relevant databases, PubMed, LILACS, CINAHL, SCOPUS and Google Scholar will be searched from 1^st^ January 2000 to 31^st^ March 2024 using search terms developed from the major concepts in the title without restrictions by language. The Cochrane Library, African Journals Online, Data Base of African Thesis and Dissertations Including Research (DATAD-R D Space) and preprint repositories will also be searched. Reference lists of relevant studies will be searched and experts in the field will be contacted for additional (unpublished) studies. The search output will be exported to Endnote where duplicate studies will be removed. The deduplicated studies will be exported to Rayyan where study screening and selection will be conducted. At least two authors will independently select studies, extract data and assess quality in the included studies using pretested tools. Disagreements between reviewers will be resolved through discussion. Data analysis will be performed with RevMan 5.4. Comparative binary outcomes will be reported as odds ratio (OR) or risk ratio (RR) and for continuous outcomes, mean difference and standard deviations (SDs) will be used. Non-comparative studies will be analysed as weighted proportions. Heterogeneity between studies will be assessed graphically and statistically, and where a significant level is detected, the random-effects model meta-analysis will be performed. All estimates will be reported with their 95% confidence intervals (CIs). Where data permit, we will conduct subgroup and sensitivity analyses to test the robustness of the estimates on key quality domains. The overall quality of the evidence will be assessed using GRADE (Grading of Recommendations Assessment, Development and Evaluation).

**Expected study outcomes:**

This systematic review and meta-analysis uses rigorous methods and best practices to attempt to collate all empirical evidence and estimate country-specific proportions of iatrogenic (surgery-related) fistulas among obstetric fistula patients across countries in SSA. This review will explore context-specific variables, provide insights into their impact and relate them to the type and experience of personnel performing the obstetric procedures that lead to obstetric fistulas. The findings of the full review are expected to inform the development of national and regional Training Programs for Medical Officers, support the development of a consensus “minimum acceptable standard of care” and inform quality assurance standards for clinicians involved in the provision of surgical obstetric care.

## Background

Obstetric fistulas, abnormal connection(s) between the vagina and the urinary tract (urethra, bladder or rarely ureters) or the rectum, resulting in urine and/or faecal incontinence, are serious childbirth injuries sustained by mothers [1, 2]. The main cause of fistula development is obstructed labour [3] where prolonged pressure of the foetal presenting part (often the head) against the pubic bone compromises blood supply in the interposing vaginal and lower urinary tract tissue leading to tissue death, sloughing and fistula formation. Fistulas may also occur as complications of surgery (iatrogenic fistulas) [4–7]. A systematic review involving 15 studies published between 2010 and 2020 reported gynaecological surgery, obstructed labour, and caesarean section as the major causes of vesicovaginal fistulas [8].

Over 2 million women of reproductive age suffer obstetric fistulas, with thousands of new cases every year [9]. Most obstetric fistulas occur in SSA and Asia [10] where health systems are weak and failures to provide universally accessible, timely and quality obstetric care are common [11]. Europe and the United States of America have been able to address the fistula problems since the 1950’s and this has been attributed to the availability of universal access to quality healthcare delivery and emergency obstetric services [12]. However, this is still a major problem in SSA with shortage of trained staff, lack of or limited medical supplies, poor quality of care, long waiting times, poor referral systems and poor coordination of tasks among staff [13]. The distance pregnant women have to travel to access the nearest health facility, poor road networks and high transport costs further complicate the situation, limiting pregnant women’s access to quality care during childbirth across countries in SSA [14, 15]. Other predisposing factors to obstetric fistulas are small pelvis, foetal mal-presentations or abnormalities, duration of obstructed labour and therapeutic misadventure [16, 17]. Early child bearing [15, 18], low educational levels [19–21] and poverty [22], as well as cultural factors such as female genital mutilation [23] and the patrilineal system that maintains male authority in the decision making process for obtaining healthcare influence health seeking behaviour and contribute to the increased risk of fistulas [24].

The effort to reduce maternal mortality and increase access to emergency obstetric care has resulted in increased caesarean section rates [25] which in turn has led to more surgery-related urogenital fistulas [26]. Although mostly occurring in the context of protracted labour, iatrogenic fistulas have been increasing in elective caesarean sections [27]. This raises serious concerns about the quality of obstetric care in SSA [28] and the training needs of healthcare professionals across these countries in the south of the Sahara [29]. A multi-country study involving sub-Saharan African countries observed that over 90% of obstetric surgeries resulting in fistula were performed by non-specialists [27]. This is as a result of widespread surgical task shifting, commonly employed in low- and middle-income countries (LMIC’s) in attempts to mitigate the critical shortage of Specialist Surgical Workforce [30–32]. Currently, there are less than 3 specialists per 100,000 population in most SSA countries [33] of whom only a fraction are qualified obstetricians. Obstetric fistula prevention is an integral component of the sustainable development goals (SDGs) 3 and 5.6 –ensuring healthy lives for all and promoting universal access to sexual and reproductive health by 2030 [11]. It is, therefore, imperative that urgent effort be directed towards reducing and/or preventing this tragic consequence of childbirth in vulnerable pregnant women [34].

To justify our systematic review is not duplicating existing reviews, we conducted searches in relevant databases (described in the methods of the abstract and main document) and retrieved eleven reviews [8, 35–44] on fistulas. Of these, five had a global focus [8, 35, 37–38, 44], three focused on Low and Middle-Income Countries (LMICs) [36, 39, 43] and three, Africa [40–42]. None of the systematic reviews covering LMICs or Africa focused on the issues our systematic review aims to investigate. Of the reviews having global focus, three [8, 35, 38] were somehow aligned with our review but two were published in 2013 and 2016 [35, 38] and are outdated, whereas the only relevant review [8] targeted only a single type of obstetric fistulas, vesicovaginal fistula.

This systematic review seeks to determine the magnitude of surgery-related obstetric fistulas in SSA and relate the estimates to the type and experience of personnel (specialist or non-specialist) performing the surgical operations. The findings and conclusions will help inform sound policies, programs and human resource planning aimed at addressing the increasing levels of iatrogenic fistulas in SSA.

## Methods

The Preferred Reporting Items for Systematic Review and Meta-Analyses extension for Protocols (PRISMA-P) (S1 Table) [45] will guide the reporting of this protocol and the PRISMA flow diagram (S1 Fig) [46] will guide the study selection process. The full review will be prepared in line with the Preferred Reporting Items for Systematic Review and Meta-Analysis (PRISMA) [47]. The protocol for this systematic review and meta-analysis is registered in the International Prospective Register for Systematic Reviews (PROSPERO), with registration ID CRD42021277993.

### Criteria for considering studies for this systematic review

#### Types of studies

Any study, including randomized controlled trial (RCT), quasi-RCT, cohort, case-control and cross-sectional studies will be eligible for inclusion in this review. Studies using secondary data, commentaries, editorials, opinions and country level statistical reports will not be eligible for inclusion. Case studies and case series will not be eligible for inclusion because these are atypical and not representative of the source population. This study will not incorporate reviews, as the unit of analysis will be restricted to primary studies. Nevertheless, we will carefully examine the reviews to identify any potentially eligible studies that may not have been captured in our searches. If the study is a global review having, for example, SSA as a sub-set or sub-regional focus, such a review will not be included as a whole. Instead, we will retrieve studies conducted in SSA and assess for inclusion. If the study reported a country or regional estimate without a well-defined representative sample or sub-sample within the source population, it will not be eligible for inclusion. For a multi-country study that included studies from SSA and reported data separately for each country, data from the SSA country will be included. In cases where the results have been lumped together and there is no way of disaggregating the data, such studies will not be included. Commentaries or opinions will not be eligible for inclusion.

#### Types of participants

The review will include women living in Sub-Saharan Africa (SSA) with urogenital or rectovaginal fistulas resulting from childbirth complications due to symphysiotomies, episiotomies, operative vaginal deliveries (forceps or vacuum delivery), caesarean delivery, caesarean hysterectomy or laparotomy due to ruptured uterus and were co-included with the aforementioned, through vaginal deliveries. Women with urogenital or rectovaginal fistulae resulting from assault, gynaecological surgeries, malignancies or radiation will not be considered, except when reported concurrently with obstetric fistulas.

#### Intervention/exposure

The exposure of interest is obstetric surgery including caesarean section, laparotomy for repair of raptured uterus, subtotal hysterectomy or hysterectomy for obstetric reasons such as raptured uterus and intractable postpartum haemorrhage.

#### Controls

Controls will consist of women with urogenital or rectovaginal fistulas resulting from vaginal deliveries without any obstetric surgery.

#### Outcomes

*Primary outcome*.

- Proportion of iatrogenic obstetric fistulas among obstetric fistula patients measured as:
$$\frac{Total\;number\;of\;obstetric\;fistulas\;arising\;from\;obstetric\;surgeries\;}{Total\;number\;of\;obstetric\;fistulas}x\;100$$
- Proportion of iatrogenic obstetric fistulas among genitourinary/rectovaginal fistula surgeries measured as:
$$\frac{Total\;number\;of\;obstetric\;fistulas\;arising\;from\;obstetric\;surgery}{Total\;number\;of\;urogenital\;and\;rectovaginal\;fistulas}\;x100$$

*Secondary outcomes*.

- Personnel performing obstetric surgeries i.e. Clinical Officers/Medical Licentiates, General Medical Doctors/Practitioners, Registrars and Specialists Obstetrician-Gynaecologists
- Correlates of surgery related obstetric fistulas amongst women in SSA

#### Search methods for identification of studies

Studies (published and unpublished) from a wide range of sources will be retrieved and assessed for eligibility. All relevant databases, including PubMed, LILACS, CINAHL, SCOPUS and Google Scholar will be searched from 1^st^ January 2000 to 31^st^ March 2024 without language restriction using search terms developed from the inclusion/exclusion (Table 1). We will also search African Journals Online (AJOL), Cochrane Library, Data Base of African Thesis and Dissertations Including Research (DATAD-R D Space) and preprint repositories for additional studies. Conference proceedings will be searched, and the reference lists of all potentially relevant studies will be checked to retrieve studies missed by our searches. Where necessary we will contact experts in the field across Africa and those affiliated to the International Society of Obstetric Fistula Surgeons (ISOF) and the International Federation of Gynaecologists and obstetricians (FIGO) via emails, and where necessary phone calls, to see if they have knowledge about any study (unpublished) we could include in the systematic review.

**Table 1:** Search strategy developed for PubMed.

Search	Query	Results
1	“Obstetric fistula” OR “Vesicovaginal fistula” OR “Vesico-vaginal fistula” “Vesicovaginal fistulae” OR “Vesico-vaginal fistulae” VVF OR “Rectovaginal fistula” OR “Recto-vaginal fistula” OR “Rectovaginal fistulae” OR “Recto-vaginal fistulae” OR RVF OR “Urogenital fistula” OR “Urethrovaginal fistula” OR “Urethrovaginal fistulae” OR “Urethro-vaginal fistula” OR “Urethro-vaginal fistulae”	
2	Iatrogenic OR “surgery related” OR “caesarean section” OR “caesarean delivery” OR “caesarean hysterectomy” OR “emergency hysterectomy” OR “Subtotal Hysterectomy”	
3	“Sub Saharan Africa” OR “Sub Saharan African” OR SSA OR Africa OR African OR Angola OR Benin OR Botswana OR “Burkina Faso” OR Burundi OR Cameroon OR “Cape Verde” OR “Central African Republic” OR Chad OR Comoros OR Congo OR “Congo Democratic Republic” OR “Cote d’Ivoire” OR Djibouti OR “Equatorial Guinea” OR Eritrea OR Eswatini OR Ethiopia OR Gabon OR Gambia OR Ghana OR Guinea OR”Guinea Bissau” OR “Ivory Coast” OR Kenya OR Lesotho OR Liberia OR Madagascar OR Malawi OR Mali OR Mauritania OR Mozambique OR Namibia OR Niger OR Nigeria OR Reunion OR Rwanda OR “Sao Tome and Principe” OR Senegal OR Seychelles OR “Sierra Leone” OR Somalia OR “South Africa” OR “South Sudan” OR Tanzania OR Togo OR Uganda OR “Western Sahara” OR Zambia OR Zimbabwe OR “West Africa” OR “West African” OR “Western Africa” OR “East Africa” OR “East African” OR “Eastern Africa” OR “Central Africa” or “Central African” OR “Southern Africa” OR “Southern African”	
4	((#1) AND (#2))	
5	((#1) AND (#2)) AND (#3).	

### Managing the search output and selecting studies

**Fig 1:**
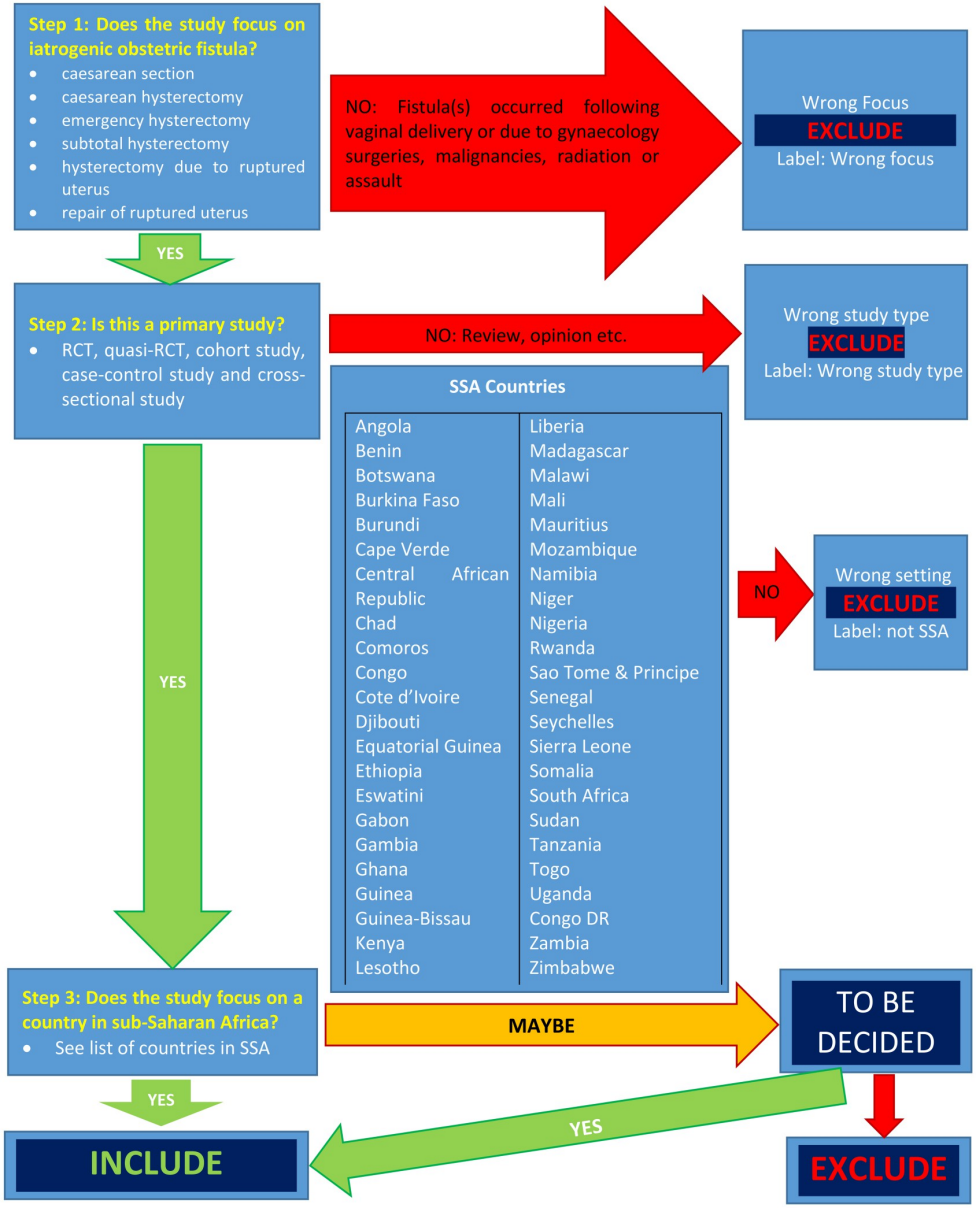
Study selection flow chart.

The result from the databases will be exported to Endnote and duplicates removed. The deduplicated studies will be exported to Rayyan software [48] for screening and selection using a study selection flowchart (Fig 1). At least two authors will independently screen the titles and abstracts of retrieved articles and select studies. The full text of all potentially eligible studies will be retrieved and assessed for inclusion. The flow of studies through the selection process will be presented using the PRISMA flow diagram (S1 Fig). Any disagreements will be resolved through discussion between the review authors.

#### Assessment of quality of the included studies

For observational studies, we will assess the risk of bias in the included studies using the quality assessment tool developed by Hoy *et al*. [49] (S2 Table). The tool assesses 10 domains, namely, representation, sampling, random selection, non-response bias, data collection, case definition, reliability tool, prevalence period, numerators and denominators. The first four domains assess the external validity in the included studies, whereas the remaining domains (5–10) assess internal validity. Responses to each of the 10 criteria on the tool will be judged as ‘low’, ‘high’ or ‘unclear’ risk of bias. The Cochrane risk of bias (RoB 2) tool will be used to assess risk of bias in the included RCTs [50] (S3 Table). This tool consists of seven evidence-based criteria namely, sequence generation, allocation concealment, blinding of participants and personnel, blinding of outcome assessors, incomplete outcome data, selective outcome data reporting and other biases. Responses to each criterion will be judged as ‘low’, ‘high’ or ‘unclear’ risk of bias. Each study’s overall risk of bias will be assessed by combining the risk of bias of the seven domains and judged as ‘low’, ‘high’ or ‘unclear’. If necessary, we will add the quality assessment tool developed by Munn et al. [51] (S4 Table). The tool is used to assess the internal and external validity of a wide range of study designs that contain prevalence data. It assesses nine quality domains: representativeness of the sample, appropriateness of recruitment, adequacy of sample size, appropriateness of descriptions and reporting of the study subjects and setting, as well as data coverage of the identified studies. The tool also addresses reliability and objectivity of the condition measured, appropriateness of statistical analyses and accountability for confounder, subgroups and differences. Each domain on the tool is assessed and assigned “yes”, “no”, “unclear” or “not applicable”. Two reviewers will independently assess quality of the included studies, any differences in responses will be discussed and where necessary a third reviewer will be engaged to resolve disagreements. The overall rating of risk of bias will be based mainly on the internal validity domains and rated as ‘low’ or ‘high’ quality using the Grading of Recommendations Assessment, Development and Evaluation (GRADE) approach.

#### Data extraction and management

Data will be extracted using a pre-tested data extraction sheet developed in Excel. The following data will be extracted: study ID, country study was conducted, year study was conducted, study design, method of data collection, eligibility criteria and sample size. We will also extract data on criteria for diagnosis, types of fistulas, cause of fistulas, mode of delivery and foetal outcomes in antecedent pregnancy, number of iatrogenic obstetric fistulas, total number of obstetric fistulas and genitourinary/rectovaginal fistulas. Health systems variables such as level of expertise of the operating surgeon (non-specialists: Clinical Officer, Medical Officer, General Practitioner/ General Medical Officers; and Specialists: Registrar(s) or Senior Registrar/Consultant Obstetrician Gynaecologists). At least two reviewers will extract data independently and resolve discrepancies through discussion.

#### Data synthesis and assessment of heterogeneity

Review Manager will be used to run statistical analyses. Binary/dichotomous outcomes will be measured as odds ratio (OR) or risk ratio (RR) and continuous data will be measured as mean difference (MD), each will be reported with their 95% confidence intervals (CIs). Heterogeneity will be assessed at three levels, clinical, methodological, and statistical heterogeneity. For the assessment of clinical heterogeneity, we will explore differences between study characteristics such as the study populations, interventions, and outcomes. Methodological heterogeneity will explore differences between studies in terms of their design and quality dimensions whereas for statistical heterogeneity, we will assess the variation of effects between studies by inspecting the forest plots for overlapping CIs and conducting statistical tests (chi-squared test and I-squared statistic (I^2^). Potential sources to be explored for the presence of heterogeneity in the present systematic review include, but not limited to, differences in study designs and country where study was conducted as it is anticipated that there will be differences in systems and resources (human and material) in different countries and settings. Additionally, differences in fistula types, type of personnel conducting the surgery and type of surgery antecedent to obstetric fistula development, which are potential sources of heterogeneity, will form the basis for subgroup analyses. The I^2^ statistic will be used to measure the extent of heterogeneity across the studies in the meta-analysis [52]. The studies will be considered to have a low level of heterogeneity if I^2^ is ≤ 25%, moderate heterogeneity when I^2^ is 26–50% and high level of heterogeneity if I^2^ > 50% to 75% [53, 54]. A random-effects model will be employed in the case where heterogeneity between studies is appreciable otherwise a fixed-effect model will be used where heterogeneity is low to moderate [55]. Subgroup analysis will be employed to address heterogeneity.

#### Dealing with missing data

We will not impute data when addressing missing data but instead we will contact primary study authors and ask for the raw data, if possible, to enable us to extract the missing information. When it is not possible to obtain missing data, only records with complete data on the outcome will be included i.e. complete case analysis.

#### Ethics and dissemination

The study does not require ethical clearance as it involves the use of secondary data. The results of the systematic review and meta-analysis will be shared with stakeholders, presented at scientific conferences and published in a peer-reviewed journal. The findings will also be shared on other public platforms such as Twitter, LinkedIn, and WhatsApp.

## Discussion

Ending obstetric fistulas is a public health and human rights priority [11]. However, if iatrogenic fistulas continue to occur at present rates, a substantial caseload of fistulas will remain for years to come, even if fistulas from prolonged obstructed labour are eliminated [56]. The shift in the cause of obstetric fistulas from obstructed labour to iatrogenic injury raises serious concerns about the quality of obstetric operative care. This study will provide insights into the burden of iatrogenic obstetric fistulas and type of personnel performing surgeries preceding fistula development. The systematic review findings will be useful for informing training program standards for medical officers contribute to the development of a consensus “minimum acceptable standard of care” and inform quality assurance standards for clinicians involved in the provision of surgical obstetric care. Additionally, the gaps identified from the systematic review will inform the scientific community on research priorities tailored towards reduction of preventable maternal morbidity.

### Study limitations

The anticipated low quality of the individual primary studies that will be included may affect the quality of evidence generated from the review. We will attempt to address quality-related limitations by conducting risk of bias assessment using validated tools specified in the text. There is also the potential for publication bias that may result from under-reporting of studies with negative findings. Gray literature and Dissertation databases will be explored to minimise exclusion of such evidence.

### Implications of the anticipated review findings

The estimation of burden of fistulas across countries in SSA will generate country-specific data for countries with less developed evidence synthesis expertise to have reliable evidence to inform country-specific and context-relevant policies that will enhance obstetric surgical care. The systematic review can also provide tailored evidence base that can inform standards for medical officer training programs, aid in the establishment of a universally accepted "minimum standard of care," and guide quality assurance criteria for clinicians engaged in obstetric surgical care provision. The gaps identified from the systematic review will potentially inform future research priorities tailored towards reduction of preventable maternal morbidity.

## Supporting information

S1 Fig PRISMA-P 2020 flow diagram to show studies retrieved from electronic databases and other sources for inclusion and flow to the final stage with studies included in the systematic review. (TIF)

S1 Table PRISMA-P (Preferred Reporting Items for Systematic Review and Meta-Analysis Protocols) 2015 checklist. (DOCX)

S2 Table Hoy et al. tool. (DOCX)

S3 Table Cochrane risk of bias tool. (DOCX)

S4 Table Munn et al. tool. (DOCX)
