# Supplementary figures and images for: The burden of iatrogenic obstetric fistulas in Sub-Saharan Africa: Systematic review and meta-analysis protocol
Source: PLoS One. 2024 Aug 26;19(8):e0302529. doi: 10.1371/journal.pone.0302529 (PMC11346637; doi:10.1371/journal.pone.0302529)

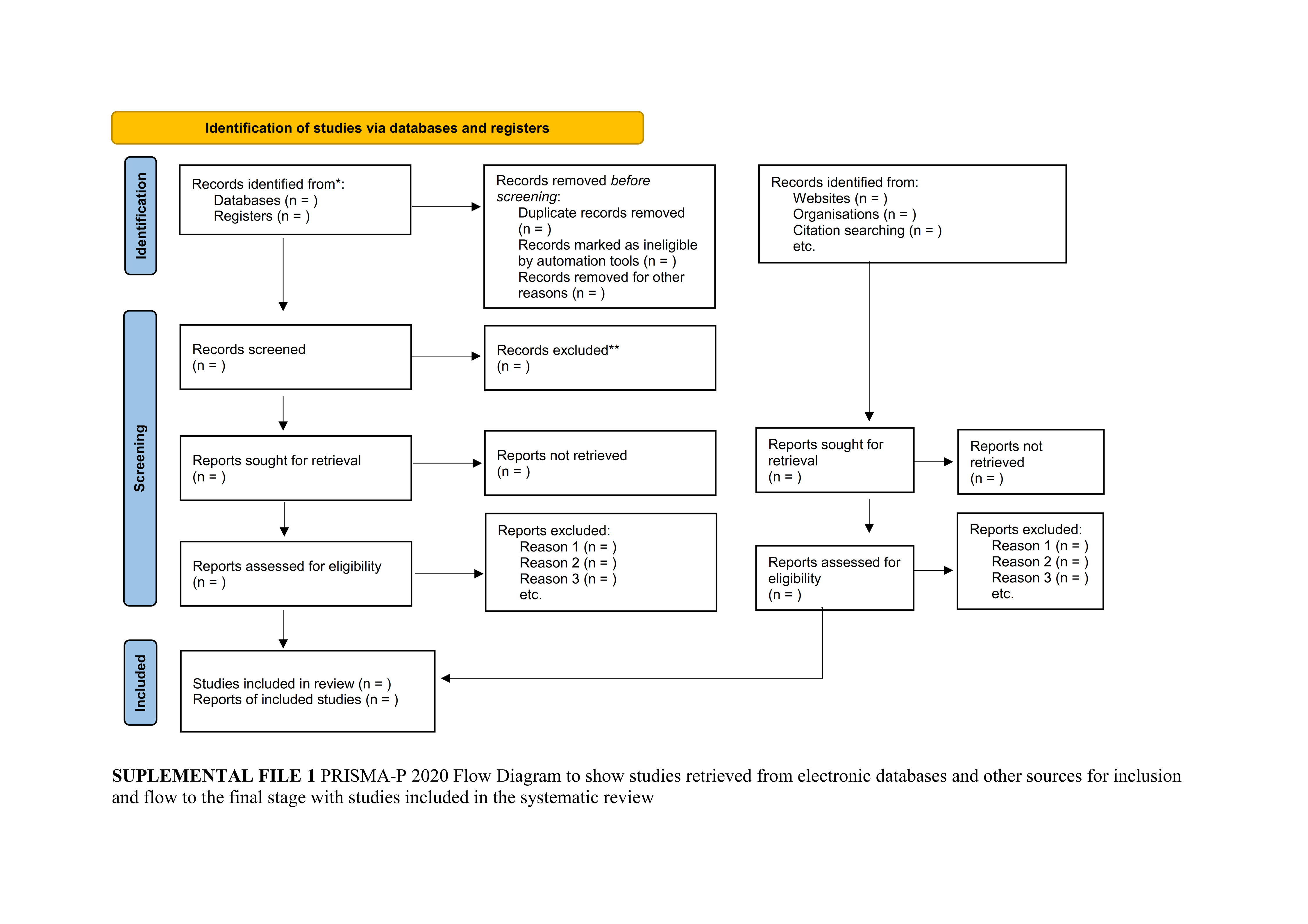

Supplement: S1 Fig — (TIF) [file pone.0302529.s001.tif]
