# Supplementary material for: The burden of iatrogenic obstetric fistulas in Sub-Saharan Africa: Systematic review and meta-analysis protocol
Source: PLoS One. 2024 Aug 26;19(8):e0302529. doi: 10.1371/journal.pone.0302529 (PMC11346637; doi:10.1371/journal.pone.0302529)
Supplement: S2 Table — (DOCX) [file pone.0302529.s003.docx]

**S2 Table. Hoy et al Risk of Bias Tool**

| **Risk of bias item** | **Criteria for answers**   - **Yes (LOW RISK)** - **No (HIGH RISK)** - **Unclear (Unclear Risk Of Bias)** |
| --- | --- |
| **External validity** | |
| 1. Was the study's target population **a close representation** of the population of interest in relation to relevant variables, e.g. age, sex, occupation, health status or other? |  |
| 2. Was the sampling frame a **true or close representation** of the target population? |  |
| 3. Was some form of **random selection** used to select the sample, OR, was a census undertaken? |  |
| 4. Did the study avoid inappropriate exclusions? |  |
| **Internal validity** | |
| 5. Was an acceptable case definition used in the study? |  |
| 6. Is the study method for measuring drug prescription shown to have **reliability and validity (if necessary)**? i.e. is there an opportunity for misclassification |  |
| 7. Was the **same mode of data collection** used for all subjects? |  |
| 8. Were the **numerator(s) and denominator(s)** for the parameter of interest appropriate? |  |
| **Summary item on the overall risk of study bias** | |
| - **LOW RISK OF BIAS:** Further research is very unlikely to change our confidence in the estimate. - **MODERATE RISK OF BIAS:** Further research is likely to have an important impact on our confidence in the estimate and may change the estimate. - **HIGH RISK OF BIAS:** Further research is very likely to have an important impact on our confidence in the estimate and is likely to change the estimate. | |
