# Supplementary material for: The burden of iatrogenic obstetric fistulas in Sub-Saharan Africa: Systematic review and meta-analysis protocol
Source: PLoS One. 2024 Aug 26;19(8):e0302529. doi: 10.1371/journal.pone.0302529 (PMC11346637; doi:10.1371/journal.pone.0302529)
Supplement: S3 Table — (DOCX) [file pone.0302529.s004.docx]

**S3 Table.** Cochrane Risk of Bias Tool

**Risk of Bias Assessment Tool**

| **Domain** | **Description** | **High Risk of Bias** | **Low Risk of Bias** | **Unclear Risk of Bias** | **Reviewer Assessment** | **Reviewer Comments** |
| --- | --- | --- | --- | --- | --- | --- |
| *Selection bias*  ***Random sequence generation*** | Described the method used to generate the allocation sequence in  sufficient detail to  allow an assessment of whether it should  produce comparable groups | Selection bias (biased allocation to interventions) due to inadequate generation of a randomized sequence | Random sequence generation method should produce comparable groups | Not described in sufficient detail | **High**  **Low**  **Unclear** |  |
| *Selection bias* ***Allocation concealment*** | Described the method used to conceal the allocation sequence in  sufficient detail to determine whether intervention allocations could have been foreseen before or during enrollment | Selection bias (biased allocation to interventions) due to inadequate concealment of allocations prior to assignment | Intervention allocations  likely could not have been foreseen in before or during enrollment | Not described in sufficient detail | **High**  **Low**  **Unclear** |  |
| *Performance bias*  ***Blinding participants and investigators**** | Described all measures used, if any, to blind trial participants and researchers from knowledge of which intervention a participant received. Provide any information relating to whether intended blinding was effective | Performance bias due to knowledge of the allocated interventions by participants and personnel during the study | Intervention allocations  likely could not have been known in, before or during the trial or participants, carers or people delivering the interventions were aware of the intervention allocation’s but no deviations from the intended intervention arose due to the trial context | Not described in sufficient detail | **High**  **Low**  **Unclear** |  |
| *Assessment/Detection bias*  ***Blinding outcome assessors**** | Described all measures used to blind outcome assessment from knowledge of which intervention a participant received. Provide any information relating to whether the intended blinding was effective | Assessment bias due to knowledge of the allocated interventions by outcome assessment | Knowledge of allocated interventions not likely to have been known prior to assessment or the assessment of the outcome could not have been influenced by knowledge of the intervention received | Insufficient information to permit judgement | **High**  **Low**  **Unclear** |  |
| *Attrition bias*  ***Incomplete outcome data*** | Described the completeness of outcome data for each main outcome including attrition and exclusions from the analysis. Stated whether attrition and exclusions were reported, the numbers in each intervention group (compared with total randomized participants), reasons for attrition or exclusions where reported, and any re-inclusions in analyses for the review | Attrition bias due to amount, nature or handling of incomplete outcome data | Outcome data were available for all, or nearly all randomized participants | There is insufficient information as to whether the results were not biased by missing outcome data | **High**  **Low**  **Unclear** |  |
| *Reporting bias*  ***Selective reporting*** | Stated how the possibility of selective outcome reporting was examined by the authors and what was found | Reporting bias due to selective outcome reporting | Selective outcome  reporting bias not detected | Insufficient information to permit judgment† | **High**  **Low**  **Unclear** |  |
| *Other bias* ***Other sources of bias*** | Any important concerns about bias not addressed above** | Bias due to problems not covered elsewhere in the table | No other bias detected | There may be a risk of bias, but there is either insufficient information to assess whether an important risk of bias exists or insufficient rationale or evidence that an  identified problem will introduce bias | **High**  **Low**  **Unclear** |  |

Bias is assessed as a judgment (high, low, or unclear) for individual elements from five domains (selection, performance, attrition, reporting, and other).

*Assessments should be made for each main outcome or class of outcomes

** If particular questions/entries were pre-specified in the study's protocol, responses should be provided for each question/entry.

† It is likely that the majority of studies will fall into this category.

Assess each main or class of outcomes for each of the following. Indicate the specific outcome.
