# Supplementary material for: The burden of iatrogenic obstetric fistulas in Sub-Saharan Africa: Systematic review and meta-analysis protocol
Source: PLoS One. 2024 Aug 26;19(8):e0302529. doi: 10.1371/journal.pone.0302529 (PMC11346637; doi:10.1371/journal.pone.0302529)
Supplement: S4 Table — (DOCX) [file pone.0302529.s005.docx]

| **S4 Table.** Munn et al Tool for studies reporting prevalence data (last amended in 2017) | | | | |
| --- | --- | --- | --- | --- |
| Major Components | Response options | | | |
| 1. Was the sample frame appropriate to address the target population? | Yes | No | Unclear | Not applicable |
| 2. Were study participants sampled in an appropriate way? | Yes | No | Unclear | Not applicable |
| 3. Was the sample size adequate? | Yes | No | Unclear | Not applicable |
| 4. Were the study subjects and the setting described in detail? | Yes | No | Unclear | Not applicable |
| 5. Was the data analysis conducted with sufficient coverage of the identified sample? | Yes | No | Unclear | Not applicable |
| 6. Were valid methods used for the identification of the condition? | Yes | No | Unclear | Not applicable |
| 7. Was the condition measured in a standard, reliable way for all participants? | Yes | No | Unclear | Not applicable |
| 8. Was there appropriate statistical analysis? | Yes | No | Unclear | Not applicable |
| 9. Was the response rate adequate, and if not, was the low response rate managed appropriately? | Yes | No | Unclear | Not applicable |
